# Progress of Interfacial Polymerization Techniques for Polyamide Thin Film (Nano)Composite Membrane Fabrication: A Comprehensive Review

**DOI:** 10.3390/polym12122817

**Published:** 2020-11-27

**Authors:** Mei Qun Seah, Woei Jye Lau, Pei Sean Goh, Hui-Hsin Tseng, Roswanira Abdul Wahab, Ahmad Fauzi Ismail

**Affiliations:** 1 Advanced Membrane Technology Research Centre (AMTEC), Universiti Teknologi Malaysia, Johor Bahru 81310, Malaysia; mei.qun-1995@graduate.utm.my (M.Q.S.); peisean@petroleum.utm.my (P.S.G.); afauzi@utm.my (A.F.I.); 2 Department of Occupational Safety & Health, Chung Shan Medical University, Taichung 40201, Taiwan; 3 Department of Occupational Medicine, Chung Shan Medical University Hospital, Taichung 402, Taiwan; 4 Department of Chemistry, Faculty of Science, Universiti Teknologi Malaysia, Johor Bahru 81310, Malaysia; roswanira@kimia.fs.utm.my

**Keywords:** interfacial polymerization, polyamide, thin film composite, membrane, nanomaterials

## Abstract

In this paper, we review various novel/modified interfacial polymerization (IP) techniques for the fabrication of polyamide (PA) thin film composite (TFC)/thin film nanocomposite (TFN) membranes in both pressure-driven and osmotically driven separation processes. Although conventional IP technique is the dominant technology for the fabrication of commercial nanofiltration (NF) and reverse osmosis (RO) membranes, it is plagued with issues of low membrane permeability, relatively thick PA layer and susceptibility to fouling, which limit the performance. Over the past decade, we have seen a significant growth in scientific publications related to the novel/modified IP techniques used in fabricating advanced PA-TFC/TFN membranes for various water applications. Novel/modified IP lab-scale studies have consistently, so far, yielded promising results compared to membranes made by conventional IP technique, in terms of better filtration efficiency (increased permeability without compensating solute rejection), improved chemical properties (crosslinking degree), reduced surface roughness and the perfect embedment of nanomaterials within selective layers. Furthermore, several new IP techniques can precisely control the thickness of the PA layer at sub-10 nm and significantly reduce the usage of chemicals. Despite the substantial improvements, these novel IP approaches have downsides that hinder their extensive implementation both at the lab-scale and in manufacturing environments. Herein, this review offers valuable insights into the development of effective IP techniques in the fabrication of TFC/TFN membrane for enhanced water separation.

## 1. Introduction

Thin film composite (TFC) membranes are the dominant technology for the commercial market of nanofiltration (NF) and reverse osmosis (RO) process. Compared to the microporous membranes, the TFC-NF and -RO membranes show better separation efficiency in producing high-quality water, as a result of their dense skin layer made of a crosslinked polyamide (PA) network [1]. Generally, TFC-NF membranes are used in water purification, wastewater treatment, pharmaceutical and biotech industries, among others [2], while TFC-RO membranes are mainly for brackish water and seawater desalination process [3]. In 2017, the global NF and RO membrane market was valued at $643 million and $6.9 billion, respectively, and was projected to reach $955 million and $13.5 billion by 2025 [4,5]. The estimated compound annual growth rate of over 5.0% for the period between 2018 and 2025 reflects the ever-increasing potential of TFC membranes for industrial applications.

In the 1950s, Loeb and Sourirajan invented the first polymeric membrane made of cellulose acetate with ~99% rejection efficiency for removing dissolved ions from seawater [6]. Nevertheless, its application was hampered by low water permeability (~0.14 L/m^2^·h·bar) coupled with poor chemical and pH tolerance. Furthermore, cellulose-based membranes also exhibited low temperature resistance that rendered them incompatible for use at elevated temperatures [7]. The cellulose-based membranes for desalination were soon phased out after the TFC membrane was developed by Cadottee and his colleagues in the 1970s [8]. This composite membrane was produced by depositing thin PA selective layer over a microporous membrane substrate via the interfacial polymerization (IP) technique. Aside from showing better chemical and pH tolerance, the TFC membrane demonstrated a similar salt rejection efficiency with an added advantage of a higher water permeability (~0.74 L/m^2^·h·bar) than cellulose-based membrane, when it was first reported.

**Figure 1:**
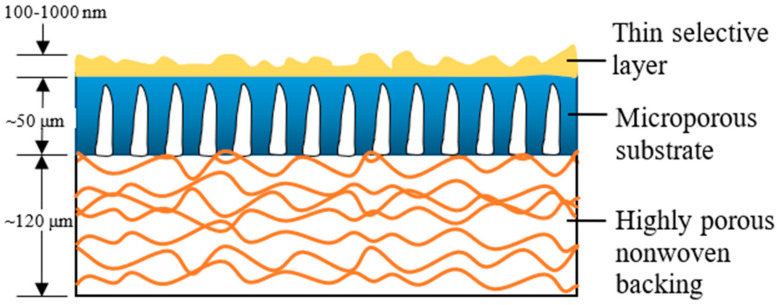
Schematic cross-section of a thin film composite (TFC) membrane and typical characteristics of each layer.

Over the past two to three decades, the TFC membrane perhaps is the fastest-growing membrane technology for the treatment of industrial water and wastewater [5], as its properties (PA layer and substrate) can be independently optimized to achieve the desired separation performance [9]. Figure 1 illustrates the typical construct of a commercial TFC membrane showing three distinct layers, i.e., the top PA layer (responsible for the membrane selectivity) supported by microporous substrate and thick polyester nonwoven backing. The bottom polyester layer is particularly important for mechanically supporting the entire membrane sheet to withstand high-pressure filtration. Although TFC membranes are widely used in many industries; without facing major technical issues, their performances are far from ideal with the major hurdle being the trade-off effect between membrane water permeability and salt rejection [10,11,12], alongside vulnerability to organic/inorganic fouling, as well as chlorine attack.

Concerted efforts into improving membrane characteristics and consequently filtration performance have generally centered on integrating inorganic nanomaterials to either the PA layer or microporous substrate [13,14,15]. The resultant thin film nanocomposite (TFN) membrane has since become popular among membrane scientists, from the time when it was first reported in 2007, by Jeong et al. [16]. Compared to the use of nanomaterials to modify substrate, utilizing nanomaterials for PA layer modification has greater significance for water application, as it is the layer that is directly exposed to the feed solution. While there are several comprehensive reviews available on this topic [17,18,19,20,21], they largely cover the impacts of different nanomaterials on the physiochemical properties of TFN membrane and how changes in membrane intrinsic characteristics could affect membrane performance.

Although laboratory-scale studies generally yielded promising results after the PA layer is modified by nanomaterials, a larger-scale TFN membrane manufacture remains challenging. It has to do with the problematic development of defect-free PA layer for long-term operation, resulting from the poor compatibility between the polymer and the inorganic nanomaterials as well as their uneven distribution within the thin PA layer [22,23,24]. The loss of precious nanomaterials during IP processing and/or their leaching from TFN membrane during filtration are also major concerns for their industrial implementation [25]. To address these issues, modification on the conventional IP procedure is required to improve the surface properties of the PA-nanomaterials layer. There has been a significant number of modified IP processes or newly developed IP techniques used for the TFN membrane fabrication over the last decade [26,27,28]. These include the filtration-based IP [29] and spin-based IP [30]. In certain cases, the water fluxes of the resultant membranes fabricated by modified or new IP technique were reported to increase by an order without compromising salt/solute rejection, in comparison to the conventionally prepared TFC membrane [31,32,33].

Modifications of conventional IP technique for developing extremely thin PA layer for ultrafast solvent permeation have also been described. Karan et al. [32] reported that a high permeation rate sub-10 nm PA layer could be synthesized by using a controlled IP technique, by sacrificing a nanostrand interlayer on the substrate. Conversely, Park et al. [34] found that the use of support-free IP technique could facilitate better characterization of the PA layer structure through easier isolation. It is apparent that such promising results could not be achieved without modifying the conventional IP technique.

In this article, we intend to review the previously reported novel/modified IP techniques for the fabrication of TFC/TFN membranes for the different domains of application comprising of NF and RO process, as well as the osmotically driven membrane process, namely, forward osmosis (FO) and pressure retarded osmosis (PRO). Seven main novel/modified IP techniques are reviewed, and their efficiencies are examined by comparing with the conventional IP process. Lastly, the technical challenges of each novel IP technique are also discussed to provide a clearer insight into their practicality.

## 2. Conventional Interfacial Polymerization Technique

**Figure 2:**
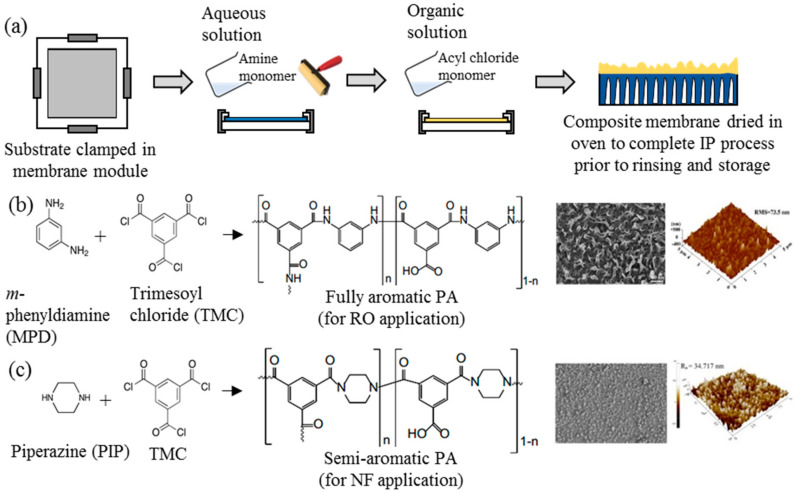
(**a**) Typical lab-scale polymerization (IP) process for TFC flat sheet membrane fabrication and (**b**,**c**) the two most common crosslinked polyamide (PA) structures for commercial TFC nanofiltration (NF) and reverse osmosis (RO) membranes and their respective FESEM and AFM surface images [40,41].

Ever since its discovery, IP has been an important process in the generation of thin active layer to produce of both NF and RO membranes [35,36,37,38,39,40]. This process establishes a highly crosslinked PA active layer on the surface of a microporous substrate through copolymerization between two immiscible reactive monomers in different medium, i.e., aqueous and organic phase [18]. Figure 2a illustrates the typical procedure of TFC membrane fabrication via the IP technique. The substrate is immersed into an aqueous solution containing the amine monomer before encountering a secondary monomer in an organic solution. In most cases, the *m*-phenyldiamine (MPD) concentration (for RO membrane fabrication) is often reported to be 2 wt % [38,41,42,43,44,45,46,47], while the piperazine (PIP) concentration (for NF membrane fabrication) ranges between 1 and 2 wt % [35,37,48,49,50,51,52]. For the secondary monomer solution, the trimesoyl chloride (TMC) concentration is normally kept at lower range (0.1–0.2 wt %) for both RO and NF membrane fabrication. The higher amine-to-acyl-chloride ratio is preferred, to ensure a complete polymerization, while simultaneously preventing acyl chloride hydrolysis that can hinder the formation of amide bonds, and lower the crosslinking degree of the polymer network [53]. Once both monomers react, a crosslinked PA network would be formed. The resultant TFC membrane is then subjected to heat treatment (60–80 °C) to complete the film polymerization and to enhance adhesion between the thin PA layer and the substrate. In this case, the performance of the TFC membrane is heavily dependent on many parameters, including monomer type and concentration [54], properties of organic solution [55], polymerization reaction time [56] and temperature [42], and post-treatment conditions [42].

Figure 2b,c depicts the two commonly used monomers to prepare TFC membranes for the RO and NF process, respectively. The use of different monomers creates different surface morphologies, where the TFC membrane constructed of MPD–TMC generally exhibits a ridge-and-valley structure compared to globular structures in the membrane made of PIP–TMC. Typically, the ridge-and-valley PA layer demonstrates excellent NaCl rejection over the globular PA structure, owing to the high degree of crosslinking that produces dense skin layer [28,42]. 

Studies found that an increase in monomer concentration had a positive impact on the TFC membrane performance in terms of water flux and/or salt rejection [54,57,58], but rapidly declined when the concentration exceeded its optimum. It is difficult to precisely pinpoint the ideal concentration of monomers to be used, as there are many factors involved during the polymerization process. These include the choice of additives (e.g., triethylamine and camphorsulfonic acid) in the aqueous/organic phase [14,48,59], the monomer reaction time and temperature [60,61], properties of organic solvents [62], rinsing and drying conditions [42], as well as the employed IP method [63]. Another point to consider is the property of microporous substrate used, since any variation in its pore size, porosity, hydrophilicity and functional group can profoundly alter the formation of PA layer [31,64,65,66]. However, a comprehensive review of the substrate’s effect is beyond the scope of this current paper and readers are advised to refer to [67,68].

Although the conventional IP technique is the preferred technique to prepare commercial TFC-NF and -RO membranes, it is not without any drawbacks. For instance, the preparation of an extremely thin PA layer (e.g., <50 nm) which effectively removes ions remains a challenge [69,70,71]. Reducing selective layer thickness is critical for high water permeability of membranes and to minimize system footprint for industrial application. Another drawback of conventional IP technique is the use of either rubber roller or airgun to remove excess amine solution from the substrate. Both methods, unfortunately, negatively affect the preparation of TFC/TFN membranes in different ways, and the issues are detailed out in following subsection. 

## 3. Issues with Conventional Interfacial Polymerization Technique

**Figure 3:**
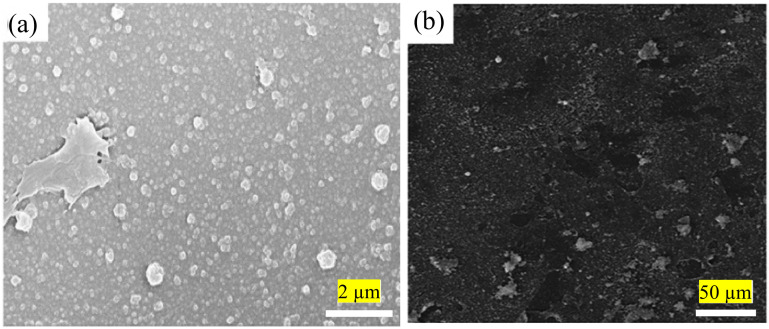
(**a**) SEM images showing agglomeration of modified-graphene oxide (GO) [48] and (**b**) multi-walled carbon nanotubes (MWCNTs) [72] in the PA layer of membrane.

Due to limitations in the current conventional IP technique that complicate the fabrication of better TFC/TFN membranes, the technique is consistently being modified to improve its performance. This is for better control of the PA formation independent of chemicals (e.g., presence of additives and/or different monomer concentrations) and thermal influence (e.g., reaction temperature and/or post-treatment parameters). The first issue with the conventional IP technique is the rubber rolling removal step that causes the amine monomer to be expelled along with the excess aqueous solution. Rubber rolling is compulsory to prevent the formation of extremely small water droplets on the substrate surface prior to the introduction of acyl chloride monomer. Without proper rolling, acyl chloride monomer would react with the water droplets (instead of amine monomer), causing lower degree of PA crosslinking and surface defects. The same method also interferes with the homogenous dispersion of nanomaterials in the PA layer during TFN membrane fabrication. This is because nanomaterials dispersed in the amine-contained aqueous solution are also removed by rubber rolling, hence affecting their distribution and, thus forming agglomeration/voids in the PA layer. Figure 3 presents SEM surface images of the TFN membranes in which nanoparticles were embedded within PA layer via conventional IP technique [48,72]. Although some studies reported that surface functionalization of the nanomaterials can reduce their agglomeration [49,52], the rubber rolling step remains the cause for the loss of precious nanomaterials during the fabrication process.

**Figure 4:**
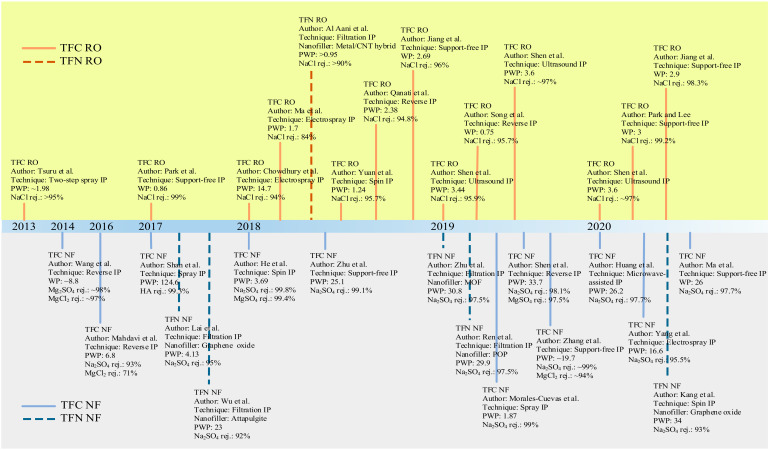
Chronological development of novel IP techniques for pressure-driven water-treatment processes. Permeability is in L/m^2^·h·bar unit.

Conventional IP process to fabricate TFC/TFN membranes also requires large quantities of organic solvents and monomers to complete film polymerization. It is the second issue plaguing this technique since excess chemicals are not reusable unless post-treatment is performed to recover them. Moreover, the seemingly low quantities of solvents and chemicals to form the crosslinked PA for a lab-scale study becomes economically unviable and non-eco-friendly at the industrial-manufacturing scale. Hence, efforts to modify conventional IP technique should also focus on minimizing chemical use. Figure 4 shows the chronological development of novel/modified IP techniques developed since 2013 for the fabrication of TFC/TFN membranes for water applications. Advancement in IP can potentially improve the intrinsic characteristics of the PA layer while offering more sustainable and environmentally friendly solutions [73].

### 3.1. Support-Free IP Technique

Support-free IP or also known as free-standing IP, is a technique that synthesizes PA layer without needing any microporous substrate, as in conventional IP. In this technique, the PA film is formed on the solution interface, floating in the excess aqueous solution before being transferred onto a substrate. Basically, the free-standing PA layer is manually lifted followed by attachment on the substrate surface [74]. This technique prevents any substrate interference which may affect the physiochemical properties of the PA layer formed. Table 1 highlights some of the important studies that work on TFC membrane fabrication, using support-free IP technique.

**Figure 5:**
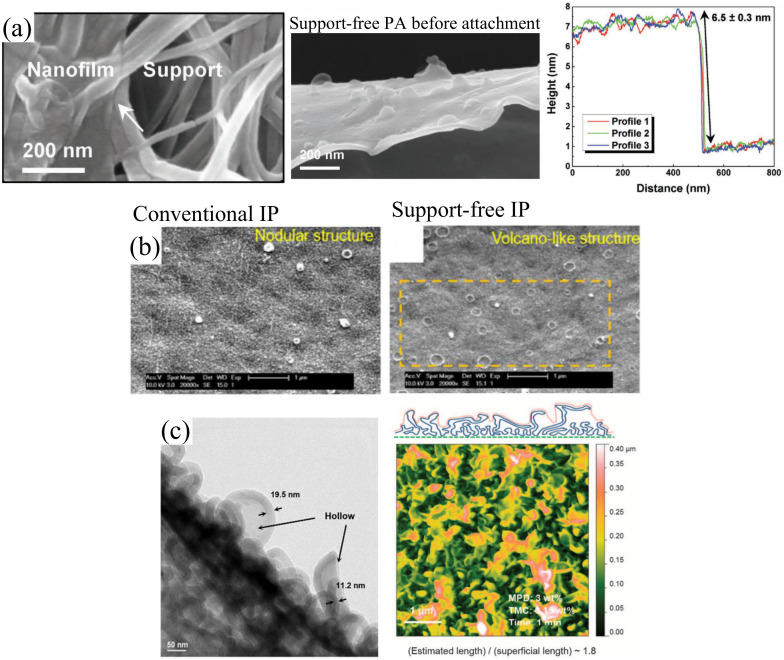
(**a**) SEM image and AFM profile of free-standing nanofilm formed without substrate interference [79], (**b**) SEM surface morphology of TFC-NF membranes formed via conventional and support-free IP [77] and (**c**) cross-sectional TEM and AFM images of crumpled PA film formed via support-free IP [32].

In 2018, Jiang et al. [79] fabricated TFC-RO membranes by forming PA nanofilm on free water–hexane interface before transferring it onto a support membrane. A sample can be seen in the SEM micrograph (Figure 5a) illustrating the firm attachment of PA onto the support membrane. Interestingly, the RO membrane formed via support-free IP showed a smooth surface with slight nodular structures, unlike the typical ridge-and-valley structures of TFC-RO membrane made by conventional IP technique. The authors attributed the smooth PA layer to the rapid heat dissipation and unobstructed release of gas nanobubbles that occurred at the free interface. A similar observation was also reported in other studies that employed the free-standing IP approach [75,80]. With respect to performance, Jiang et al. [79] reported that the newly developed membrane exhibited water permeance and NaCl rejection of 2.7 L/m^2^·h·bar and 96%, respectively, when tested by using 2000 ppm NaCl solution at 20 bar. The values were found comparable with the commercially available TFC-RO membranes. Nevertheless, taking into the account the effective permeation area of membrane, the newly developed membrane is comparatively more productive due to the relatively smaller permeation area, as a result of its smooth surface. In a separate work, Park et al. [75] reported that the support-free IP approach successfully produced an exceptional TFC membrane with a higher degree of crosslinking (O/N ratio of 1.14 compared to 0.72 in conventionally fabricated membrane) as a consequence of the enhanced and uniform amine diffusion during film formation.

With the aid of vacuum filtration, Zhu et al. [77] deposited a free-standing PA layer onto a substrate to develop TFC-NF membrane. Figure 5b compares the surface morphology of the TFC membrane made of conventional IP and free-standing IP. Interestingly, the new IP method was able to produce volcano-like structures on the PA layer, believed to have originated from eruptions of water-rich globules during the filtration procedure. Aside from being highly stable, the newly developed TFC membrane also demonstrated almost twice higher pure water flux compared to commercial membrane (NF 2A, Sepro), with the latter recording a 25.1 L/m^2^·h·bar with 99.1% Na_2_SO_4_ rejection. Similarly, Song et al. [80] drained the excess aqueous solution through the substrate by vacuum filtration, but found that the surface area was reduced due to the absence of nodules in the support-free PA. This only led to a mere 0.94 L/m^2^·h·bar in water flux, a considerable 61% reduction over the conventionally formed membrane (1.55 L/m^2^·h·bar).

The support-free IP technique is also applied in the development of advanced membranes for FO and organic solvent nanofiltration (OSN) process [32,76]. In 2016, Karan et al. [32] invented a controlled IP method by forfeiting a nanostrand interlayer to create free-standing sub-10 nm PA film for OSN application. Contrary to the expected smooth PA layer, the presence of nanostrands during PA formation produced crumpled and rigid PA textures (Figure 5c) capable of withstanding prolonged pressurized filtration (up to 9 h at 10 bar). The crumpled nanofilm provided higher permeance by a factor of >4 compared to its smoother counterpart. As a result, both acetonitrile and methanol permeance were more than two orders of magnitude higher than those of commercial OSN membranes (DuraMem DM150 from Evonik MET Ltd.) due to the increased permeable area on the crumpled nanofilm. Similar crumpled/ridge-and-valley PA structure was also reported in the work of Cui et al. [76] that aimed to develop support-free IP membranes for FO application. They left the support-free PA overnight to completely evaporate organic solvent and because of this, MPD probably continuously diffused towards the organic phase through the defects in the loose incipient film. This resulted in the rough morphology of the final membrane and achieved 2.31 L/m^2^·h·bar water flux and 96% NaCl rejection when tested under RO mode. In addition, the membrane only suffered minimal reverse salt flux (0.12 g/m^2^·h) during FO mode.

Separately, Zhang et al. [81] investigated the phenomenon of support membrane pore blockage (SMPB) during PA film polymerization, in both conventional and support-free IP techniques. The obtained SEM images showed a relatively distinct boundary between the support membrane and the PA synthesized by support-free IP technique, indicating the absence of SMPB and mechanical interlocking. Although the researchers were able to improve the water permeability of conventional membrane by ~13%, using the support-free IP technique, they later found that the PA layer could easily detach from the substrate after immersion in ethanol, hence losing its desalting ability. In contrast, the conventional membrane with SMPB demonstrated insignificant performance loss. 

**Figure 6:**
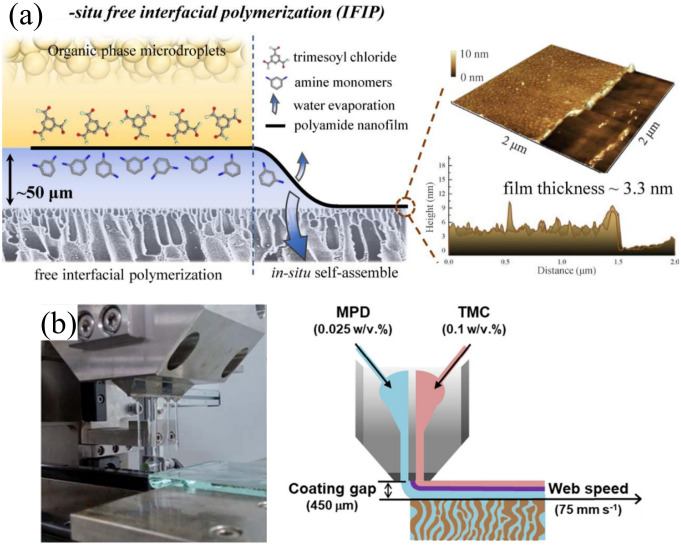
New IP technique for PA layer synthesis without having substrate. (**a**) Schematic diagram of in situ–free IP (IFIP) approach [63] and (**b**) dual-layer slot coating (DSC) apparatus and its schematic diagram [34,82].

Jiang’s group successfully fabricated high-performance TFC membranes for NF and RO application by integrating in situ IP with support-free IP; aqueous template IP (ATIP) and in situ–free IP (IFIP) [63]. In ATIP [84], the pressure-controlled rolling left a nanoscale aqueous layer on the substrate surface. Due to the presence of aqueous template, the surface morphology of the NF membrane exhibited dense ridged nanostructures with low-resistance flow channels within the ridges. This led to an exceptionally high pure water permeability (PWP) of 21.3 L/m^2^·h·bar while maintaining Na_2_SO_4_ rejection at 99.4%. The formed nanoridges were excellently stable with consistent performance even after a 200-h testing at 6 bar. This became the benchmark for the IFIP approach that was invented by the same research group later [63]. In IFIP, an aqueous layer (~50 µm) is left on the substrate (similar to ATIP) before exposure to the organic solution microdroplets (see Figure 6a), permitting the formation of a PA layer as thin as ~3–4 nm. This led to an exceptional water flux in the resultant NF (~26.6 L/m^2^·h·bar) and RO membrane (2.9 L/m^2^·h·bar), showing >3 times higher flux than the commercial NF270 and SW30XLE membranes made by using the conventional IP technique. Salt rejection in the developed membrane remained high at >98% for NF (tested with Na_2_SO_4_) and >98% for RO (tested with NaCl).

Other novel IP approaches derived from support-free IP are the dual-layer slot coating (DSC) technique developed by Park et al. [34,82] and the usage of jelly (agar hydrogel) by Ma et al. [83]. DSC enables the simultaneous and continuous spreading of two reactive monomer solutions to create an unsupported PA layer via in situ polymerization, which is then adhered onto a porous support membrane (see Figure 6b). Park et al. [34] used the DSC technique and optimized its monomer concentration to fabricate scalable TFC-RO membrane. The same research group also investigated the effect of O_2_ plasma and polydopamine coating on polysulfone (PSf) substrate prior to DSC application [82]. After substrate modification, the RO membrane permeability increased from 2 to 3 L/m^2^·h·bar without reducing NaCl rejection (>99%). The agar gel IP technique by Ma et al. [83] manipulated the hydrogel temperature to synthesize a high performance free-standing PA film at the hexane–hydrogel interface. Compared to the typical support-free PA formed at the hexane–water interface, the use of hydrogel formed a PA with 63% higher permeability (26 vs. 16 L/m^2^·h·bar) without compromising Na_2_SO_4_ rejection (97.7%). They were able to estimate the apparent activation energies required by the IP reaction by controlling the reaction temperature in the range of 0–45 °C.

Based on this review, it is apparent that the DSC, IFIP and the agar hydrogel techniques were suitable for fabricating PA thin film membranes (without using microporous substrate) with excellent performance, even at low monomer concentration (e.g., ≤0.025 wt % MPD or PIP); a feat unattainable by conventional IP technique.

### 3.2. Filtration-Based IP Technique

Vacuum filtration-based IP technique is recently found to be promising to fabricate TFN membranes by depositing a thin layer of nanomaterial on the surface of substrate via vacuum filtration prior to formation of PA layer [85,86]. Aside from the ability to evenly deposit nanomaterials on the substrate surface, this method could avoid the wastage of precious nanomaterials during the fabrication process. This IP technique eliminates the problematic rubber rolling step in conventional IP, and simultaneously averts of the loss of nanomaterials and their uneven distribution in the PA layer.

**Figure 7:**
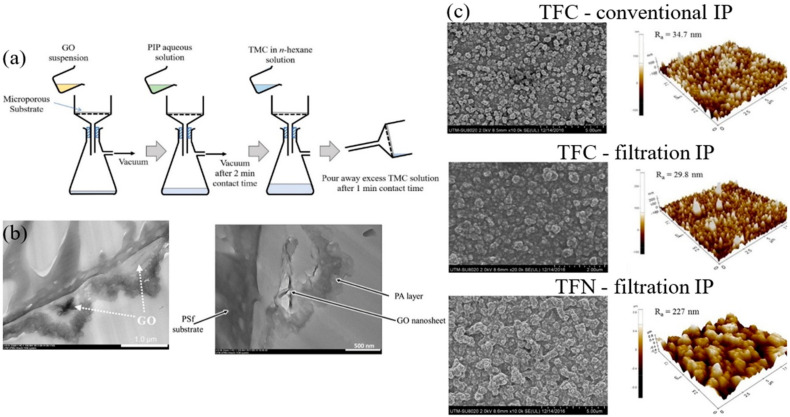
(**a**) Schematic diagram of TFN membrane fabrication via filtration IP technique [87], (**b**) TEM image of GO embedded under PA layer [28,87] and (**c**) FESEM and AFM images of composite membranes fabricated by using two different approaches [87].

Lai et al. [87] reported the suitability of the vacuum filtration-based IP technique to fabricate a new type of TFN membrane with enhanced performance for NF application. They deposited nanomaterials on the surface of microporous substrate, by vacuum-filtering aqueous solution containing GO nanosheets through the substrate. The GO-deposited substrate was then subjected to IP process by filtering a 2 wt % PIP solution followed by crosslinking with 0.2 wt % TMC (Figure 7a). The GO nanosheets were perfectly retained on the substrate surface without any loss of nanomaterials to the filtrate (Figure 7b), because the flake-form GO exhibited lateral size of several micrometers. The micrographs of the GO-deposited substrate membrane showed a rougher PA layer with coarser nodules (Figure 7c), most likely due to the interlayering of hydrophilic and rough GO that better retained excess PIP aqueous solution. The novel method could form TFC membranes with reduced PA thickness and a consequent 72% increase in PWP without any loss of Na_2_SO_4_ rejection (>95%). Anti-fouling tests using bovine serum albumin (BSA) also supported this observation as the TFC and TFN made by filtration-based IP performed better, and recorded only 16.4% and 1.1% flux decline, respectively, compared to the conventionally fabricated TFC membrane of 24.1%.

The main advantage of the filtration-based IP technique lies in its ability to remove the excess solution from the substrate surface without disturbing the nanomaterial coating that may disrupt the PA layer integrity through internal pore blockage [29]. Zhu et al. [88] explored the controlled one-step positioning of nanofillers (UiO-66-NH_2_) by filtering the PIP–nanofillers mixture through the substrate membrane and found that nanofillers could be well integrated with the selective layer. More importantly, the TFN membrane achieved PWP more than double of that of TFC membrane, recording 30.8 L/m^2^·h·bar with the Na_2_SO_4_ rejection remaining high at 97.5%. The TFN membranes also exhibited high stability with negligible performance loss for up to 180 h filtration at 4 bar, likely attributed to the firm embedment of nanomaterials within the PA layer.

A TFN NF membrane with improved water flux and rejection was also reported by Ren et al. [89] upon incorporation of *o*-hydroxy porous organic polymer in the PA layer via the vacuum filtration-based IP technique. Even without the presence of nanomaterial, they found that the TFC membrane fabricated by the filtration method showed considerably high PWP (11.5 L/m^2^·h·bar) and Na_2_SO_4_ rejection (99.2%). This was due to the unique PA morphology, i.e., crumpled, rough and thin (<90 nm). Upon embedment of 0.02 wt % nanomaterials within the PA layer, the resultant TFN membrane demonstrated an impressive water permeability of 29.9 L/m^2^·h·bar, almost triple than that the self-synthesized membrane and commercial membrane (Dow NF270).

A TFN NF membrane decorated with 0.174 mg/cm^2^ attapulgite nanorods prepared by using the filtration IP technique, improved the performance of control membrane from 17.7 L/m^2^·h·bar and 90.5% Na_2_SO_4_ rejection, to 23 L/m^2^·h·bar and 92% rejection, respectively, as described by Wu et al. [90]. The improvements were ascribed to the presence of additional hydrogen bonding between PIP and hydroxyl-rich attapulgite nanorods, leading to the formation of highly crosslinked PA with ~30% thinner structure. The embedment of nanorods within the PA layer was also stable as the membrane water flux remained consistent throughout the 50 h testing. However, it must be pointed out that nanomaterial agglomeration was likely to occur at high concentration of nanorods was used, i.e., >0.24 mg/cm^2^. A detailed comparison between the membranes formed via conventional IP and filtration IP is presented in Table 2. As can be seen, the novel technique was capable of producing membranes with superior performance and appreciable improvement in PA morphology.

### 3.3. Spin-Based IP Technique

Spin coating is a technique widely used in the microelectronics and solar cell industry to produce thin films with high uniformity [91,92], but has recently found application in fabricating antibacterial and cytocompatible membranes [93]. Aside from fabricating microporous membrane for UF application (by spin-coating polymeric dope solution directly on the disc) [94], spin-coating process has been modified from initial IP on a substrate to fabricate TFC/TFN membranes for both water [95] and non-aqueous applications [27,96], as well as gas separation [26,96].

Spin-based IP is particularly useful in forming denser PA with controllable thickness. Hence, there are many main factors governing the thickness and morphology of the PA film produced via spin coating, for instance, rotational speed and its time, including solution viscosity. Due to the spinning motion, the centrifugal shearing force acting on the microporous substrate causes the reactant to spread from the center of the substrate towards the outer edge into a thin film of uniform thickness. [95]. Table 3 compares the properties of the membranes fabricated by spin-based IP technique with the conventional IP technique over the past decade.

**Figure 8:**
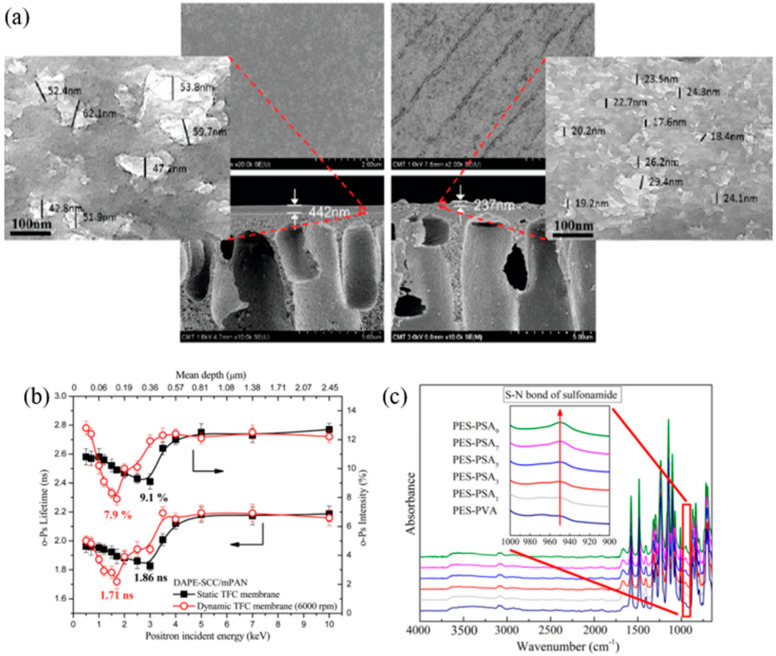
(**a**) Cross-sectional SEM images of conventional IP (left) and spin IP (right) membranes [95], (**b**) o-Ps intensity vs. positron incident energy for membranes fabricated through conventional IP and spin IP technique [95] and (**c**) ATR-IR spectra of spin-based multilayer IP (number represents deposited layer number) [97].

In 2012, An et al. [95] for the first time demonstrated the potential of integrating the IP process with spin coating, in the fabrication of TFC membrane for water-ethanol pervaporation. The centrifugal shearing force from spin-based IP process was effective in orientating polymeric molecular chains horizontally, hence forming a compact PA layer with thickness of ~237 nm, i.e., appreciably 46% thinner than the PA layer synthesized by conventional IP technique (Figure 8a). The denser PA layer led to a lower free volume intensity of 7.9%, compared to 9.1% in conventionally formed membrane, as shown in the positron annihilation lifetime spectroscopy (PALS) results (Figure 8b). The formation of the pattern lines was due to the strong shearing force contributed by the centrifugal force, causing the turbulent and unstable flow patterns of the casting solution. Using the same IP technique, Jimenez-Solomon et al. [96] fabricated an OSN membrane by forming a polyarylate polymer on top of alumina substrate. The spin-coated membrane was then tested with respect to gas permeation to confirm its enhancement in microporosity. Although the reaction between spin-coated non-conventional contorted phenols and TMC created ultrathin (~20 nm) crosslinked film, the authors were unable to prepare defect-free nanofilms large enough for lab-scale filtration tests.

Chan et al. [99] on the other hand successfully formed a PA layer with constant film growth and minimal roughness by integrating the spin coating and molecular layer-by-layer (mLbL) approach. Although the mLbL approach was similar to the traditional IP method, the spin-and-rinse-based polymerization (alternate spinning and rinsing of TMC and MPD) was able to facilitate complete reaction while maintaining monomer stoichiometry at each interface layer, resulting in the nanoscale control of PA morphology. However, the authors of this work did not perform any filtration on the developed PA film. Chan et al. [100] in separate work found that the use of this approach could lead to membrane swelling. The swelling natures of four PA layers made of different amine monomers (MPD, diethylenediamine, *p*-phenylenediamine and *o*-phenylenediamine) were modelled based on the Painter–Shenoy thermodynamic swelling models and the results showed that at higher relative humidity, the pronounced PA swelling tends to occur due to the delamination of multiple PA layers formed by using this novel method.

With the intention to develop an advanced NF membrane that is suitable for the treatment of acidic effluents, Yuan’s group [97,98] employed spin-based IP technique to fabricate TFC membrane with polysulfoneamide (PSA) as the selective layer, by sequential IP through alternate dipping and spinning of PIP and naphthalene-1,3,6-trisufonylchloride (NTSC) solution [97]. Increasing the layer number from zero to nine caused the gradual increase sulfonamide peak (950 cm^−1^) of the membrane (Figure 8c), signifying the successful PSA fabrication at each film growth. The spin-based-IP five-layer membrane achieved 96% NaCl rejection with 1.24 L/m^2^·h·bar PWP. Performance stability of the membrane was further investigated (fabricated via alternate spin-coating of PIP and tris(chlorosulfonyl)phenol (TCSP)) by immersion in acidic solution (20 wt % H_2_SO_4_) for 24 h at 90 °C [98] showed that the spin-coated five-layer membrane only experienced a minor deterioration of Na_2_SO_4_ rejection (from 99.8% to 96%). In addition, the water flux was increased by 81% compared to 115% and 155% in the conventional IP membrane made of PIP/TCSP and PIP/TMC, respectively. The lower acid stability of the two latter membranes was contributed by an enhanced dissolution of oligomers (amide bonds breakage) by sulfuric acid.

As the amount of amine monomers deposited on the substrate surface could play a significant role in influencing PA characteristics [30,84], Kang et al. [30] made an attempt to compare the effectiveness of four different removal methods, i.e., rubber-roller, vacuum filtration, spinning and gravity on the PA properties of TFC membrane. They found that spin-based removal technique was exceptional in producing TFC membrane with the highest Na_2_SO_4_ rejection (98.5%) and a relatively high PWP (28.5 L/m^2^·h·bar). Membrane PWP and salt rejection at optimal spinning conditions were able to reach ~32 L/m^2^·h·bar and ~97%, respectively. Using this technique, the monomer solution was well mixed and redistributed homogenously on the substrate by a centrifugal force that acts on the substrate in all directions. As a consequence, a PA layer with thickness of 20–35 nm was formed. Further evaluation indicated the TFC membrane made of spin-based IP technique showed not only good mechanical stability (after subject to 2 h ultrasonic treatment) but also good solvent resistances against different alcohols.

### 3.4. Ultrasound-Based IP Technique

The ultrasound-based technique is another novel IP method previously used to develop TFC membrane [101,102]. The benefits of ultrasonic waves can be found in many industrial applications including food processes (e.g., sugar crystallization, meat tenderization and drying) [103,104], clinical and medical fields [105,106,107], as well as in water treatment for membrane-cleaning processes [108,109,110]. To the best of our knowledge, only two relevant studies could be found in the literature regarding the use of ultrasound-based IP technique for TFC membrane, where both studies were done by the same research group from Huazhong University of Science and Technology, China [101]. In this method, cavitation caused by ultrasound waves can enhance the mixing efficiency at the interface area to form a looser PA layer, something impossible in the conventional diffusion-limited PA growth method.

**Figure 9:**
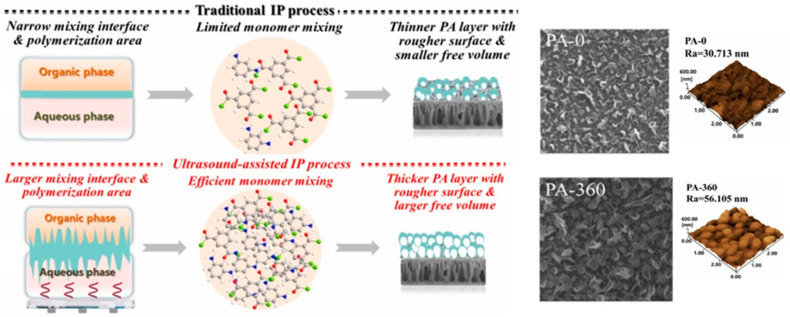
Schematic diagram, SEM and AFM images of traditional IP and UAIP process for membrane preparation (Note: PA-0—conventional TFC membrane; PA-360—ultrasonic-assisted TFC membrane [101]).

Shen et al. [101] reported that the ultrasound-assisted IP (UAIP) TFC membrane outperformed the traditional TFC membrane with respect to water flux and salt rejection. The formation of thicker but looser PA layer of ultrasound-assisted TFC membrane was the main factor leading to increase in both water flux and salt rejection (Figure 9). The degree of PA crosslinking was enhanced by the modified IP technique in which the ultrasonic waves disrupt the packing density of newly formed PA linkages, thereby enlarging aggregate voids. Another possibility has to do with the nanobubbles generated by ultrasound that was encapsulated into the PA layer, forming nanovoids. As shown in Table 4, the optimized ultrasound-assisted TFC membrane demonstrated not only good performance in the RO process (higher water flux and higher NaCl rejection) but also in osmotically driven process, i.e., FO and PRO (higher water flux, *J_v_* but reduced reverse solute flux, *J_s_*).

Shen et al. [102] further investigated the impact of ultrasonication conditions on the TFC membrane properties and reported that higher ultrasonication power coupled with higher frequency and time could increase the sonochemical effects, leading to the production of PA layer with increase in roughness, thickness and crosslinking degree. As a result, the membrane PWP was enhanced from 3.44 L/m^2^·h·bar [101] to 3.6 L/m^2^·h·bar with Na_2_SO_4_ rejection also increased from 95.9% to 97%. The UAIP-based membrane has enhanced antifouling performance against gypsum scaling, with a substantially higher FRR (97%) compared to the conventional TFC membrane (83%). This could be attributed to the highly crosslinked PA layer containing fewer carboxylate groups for interaction with scalants (CaCl_2_) in the feed solution. This, consequently, suppressed CaCO_3_-scale deposition on the membrane surface.

### 3.5. Spray-Based IP Technique

In the traditional IP process, a sprayer is used to remove the excess amine monomer solution from the microporous substrate before film polymerization takes place [37,41,111]. By modifying the traditional IP technique, a sprayer is directly employed to introduce either aqueous or organic solution (containing monomers) onto the substrate surface. Pressurized gas (air or nitrogen) ejected from an airbrush or airgun can greatly produce microscale dispersion of the monomers [112]. Bulk diffusion typically occurs in the conventional IP process followed by a stepwise diffusion that forms a dense primary and a loose secondary PA layer, respectively. With the absence of bulk solutions in the spray-based IP process, the bulk diffusion step could be eliminated which leads to the formation of a looser PA layer. Table 5 compares three relevant studies that focused on the development of TFC membranes, using spray-based IP technique.

**Figure 10:**
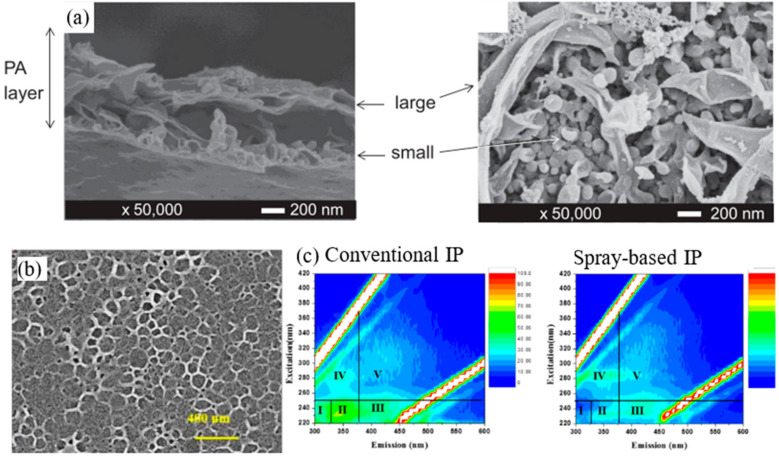
(**a**) SEM images showing large and small PA layers formed by the first- and second-step IP, respectively [112]. (**b**) SEM image of membrane prepared in high-humidity environment, resulting in the honeycomb PA structure—voids confirm the microscale monomer dispersion. (**c**) EEM analysis of permeate produced by two kinds of membranes [33].

One of the earliest mentions of the spray-based IP technique for TFC membrane fabrication originated from Tsuru et al. [112] in 2013, in which they experimented on a two-step spray-assisted IP method to produce TFC-RO membrane. Specifically, the MPD-impregnated substrate was sprayed with the TMC solution (first step) followed by drying. Then, a TMC solution of higher concentration was poured over the first-step membrane (second step), forming a multilayered structure (Figure 10a). The modified PA layer yielded homogeneously distributed small fibrous structures (resulted from spraying step) under larger leaf-like structures (resulted from pouring step). Although these fibrous structures did not contribute to any filtration performance improvement, they acted as a foundation for the second step IP, forming an ultimate PA layer with a multilayered ridge-and-valley structure. When compared to the conventional IP, the TFC membrane made of two-step spray-based IP exhibited higher PWP (1.98 vs. 1.14 L/m^2^·h·bar) with NaCl rejection remaining high at >95%. The study also reported that prolonging the spraying time from 20 to 60 s could alter the PA layer and adversely created planar structures which affected membrane surface area and reduced water permeability.

Shan et al. [33], on the other hand, employed spray-based IP to develop highly stable membrane for the removal of natural organic matter (NOM) [33]. The authors divided the bulk interface of monomers into numerous microphase interfaces in an attempt to control properties of PA layers at the nanometer scale. The approach quickly exhausted the finely dispersed reactants due to their limited amounts, forming an ultrathin PA layer (~25 nm) that was far thinner than those conventionally formed. The formed honeycomb PA structure seen in Figure 10b corresponded well to the size of sprayed droplets (<200 nm), indicating the microscale and nanoscale dispersion of the monomer solution. Further analysis revealed that the modified TFC membrane showed lower intensities in excitation emission matrix (EEM) fluorescence spectra (Figure 10c), indicating its effectiveness in micro-molecular NOM removal capacity in real water filtration.

In 2019, Morales-Cuevas et al. [113] applied spray-based IP in the fabrication of TFC-NF membranes by studying the effects of different preparation procedures, i.e., number of PA layers and moving speed of displacement device (membranes were fixed to the laterally moving displacement device facing the spray gun). The membrane with a single PA layer (made of brushing PIP followed by spraying TMC) exhibited the highest Na_2_SO_4_ rejection (99%) and relatively high permeability (1.87 L/m^2^·h·bar) compared to the PA layer fabricated via (i) spraying both PIP and TMC (1.33 L/m^2^·h·bar and 75%) and (ii) spraying PIP followed by pouring TMC (7.00 L/m^2^·h·bar and 27%). Results also indicated that the optimized spray-based TFC membrane showed 52% higher water permeability over the conventionally prepared TFC membrane (1.23 L/m^2^·h·bar). However, studies found that the spray-based approach was not feasible for the simultaneous application of both phases (i.e., spraying both PIP and TMC) due to the low wettability of the substrate [112,113], as the formed microdroplets was likely to affect the homogeneity of the resultant PA layer. In this regard, modifications to increase the substrate hydrophilicity or the integration with other techniques could be done prior to spraying.

### 3.6. Electrospray-Based IP Technique

Electrohydrodynamic process (electrospraying and electrospinning) is a technique widely used to form nanocoatings, nanoparticles and nanofibers [114,115,116,117,118]. The general mechanism lies in the ejection of a solution out of a needle due to the Coulombic repulsion force of a strong electric field, in which the technique characteristically creates a Taylor cone at the tip of the needle. In electrospraying, the electric repulsion force is useful for overcoming the surface tension of the solution so that liquid jets can be atomized into fine droplets. Meanwhile, in electrospinning, the stronger intermolecular forces in the solution results in continuous jets. Eventually, the bending and stretching during the transition of a polymeric solution to a solid state forms a nonwoven mat of nanofibers [119]. This concept is very often applied in the fabrication of microporous membranes with extremely high porosity [120,121,122].

For the synthesis of TFC membrane, there are some studies that employ electrospinning and the traditional IP to fabricate the substrate and PA layer, respectively [123,124,125]. Nevertheless, electrospraying is more suited for the non-polymeric solution (or a low elasticity solution), and often used to eject monomer solutions for the PA layer synthesis [126]. Table 6 compares the properties of TFC membranes fabricated via conventional IP and electrospray-based IP technique. It is pertinent to indicate here that parameters such as solution properties (e.g., concentration, viscosity, surface tension and conductivity), process conditions (e.g., voltage, distance and flowrate) and ambient conditions (e.g., humidity and temperature) greatly influence the physiochemical properties of the PA layer being formed by electrospraying.

**Figure 11:**
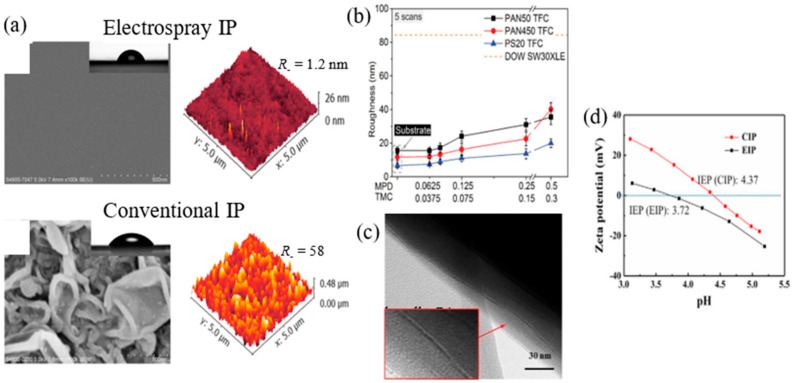
(**a**) Comparison of SEM and AFM images of TFC membrane surface formed via electrospray-based IP and conventional IP process [126]. (**b**) RMS surface roughness of electrospray-based TFC membranes fabricated at different MPD: TMC concentration ratio [31]. (**c**) TEM image showing the lamellar PA structure of electrospray IP-based membrane [127]. (**d**) Zeta potential of TFC membranes fabricated via two techniques (note: CIP, conventional IP; EIP, electrospray IP) [127].

In 2018, Ma et al. [126] reported that the PWP of TFC-RO membrane fabricated by electrospray-based IP could be enhanced by three times compared to the conventionally formed membrane, The absence of bulk solutions in the electrospray-based IP process caused the polymerization reaction to occur at the microdroplet interface, thus forming a substantially smoother surface, free of ridge-and-valley structure (Figure 11a). The technique yielded an amazingly linear growth rate of PA layer (~1 nm/min), unseen in the conventional IP process. Nonetheless, despite the excellent water permeability of the new membrane, the problem of a relatively low NaCl selectivity (84%) due to its high solute permeability coefficient (*B_NaCl_*), yet remains. Overall, its water–salt permselectivity (*A*/*B*) still falls short (0.6 bar^−1^) compared to those of commercial TFC membranes such as BW30 (>2 bar^−1^) and SW30 (>10 bar^−1^).

Chowdhury et al. [31] managed to fabricate a PA layer as thin as ~5 nm/layer via a highly regulated electrospray-based IP technique, in which the formed PA layer was subsequently attached onto the substrate to develop a RO membrane. The electrospray-based TFC membranes fabricated at different MPD/TMC concentration ratio were found to consistently exhibit smoother surface morphologies (root mean square (RMS): 7–40 nm) compared to the commercial SW30XLE membrane (RMS: >80 nm) (Figure 11b). This is a rather impressive achievement considering that even the roughest electrospray-based TFC membrane exhibited less than half of the roughness observed in the commercial membrane. The findings suggested that the electrospray-based IP approach was able to develop TFC membranes with tailorable PA thickness and intrinsic smoothness while exhibiting comparable, if not better performance. Similar membrane surface morphology was also observed in the work carried out by Yang et al. [127] on electrospray-based TFC membrane. The TEM image, as shown in Figure 11c, confirmed that the study successfully produced a PA layer showing lamellar structures as thin as 22 nm. The unique structure indicates creation of improved channels for transporting water between adjacent PA layers and, as well as an improved water permeability. As PA is grown layer by layer, the absence of the localized reaction heat in the electrospray IP process contributed to the smoother PA layer. Because of this mechanism, electrospray IP process was able to form a more negatively charged PA layer (see Figure 11d) partly due to the enhanced hydrolysis of the acyl chloride monomer during polymerization process.

With respect to filtration performance, electrospray-based TFC membranes fabricated by Chen et al. [128] exhibited high water permeance (23.7 L/m^2^·h·bar) while maintaining excellent rejection against acid yellow dye (99.2%), thus surpassing conventionally formed TFC membrane of <5 L/m^2^·h·bar usually. Uniquely, they successfully formed denser structures on the top of the looser PA layer, using different TMC concentration (0.001 wt % followed by 0.1 wt %). The dual layer PA (dense and loose layer) showed the best filtration performance (high selectivity and flux) compared to single layer of only dense or loose PA. Most importantly, the membrane with dual layer showed excellent stability in acidic conditions (pH 4), even after a 30-day testing with minimal change in dye rejection (<2.5% for all dyes tested) and water permeance (between 57 and 59 L/m^2^·h·bar). The improved structural and operation stability of the new membrane was largely due to the good interface compatibility between both layers as demonstrated by the low solvent swelling (<3% thickness swelling).

### 3.7. Reverse IP Technique

This technique was initially developed to overcome the limitations of highly hydrophobic substrates that exhibit low affinity towards water. In the reverse IP technique, the microporous substrate is first exposed to the organic solution instead of the aqueous solution, done in the conventional approach [125,129,130,131]. Studies indicated that the hydrophobic nature of the substrate prevents a uniformed dispersion of aqueous solution, hence making it unsuitable for the conventional IP approach. The consequence is seen by the formation of nonhomogeneous PA film with possible pin-hole defects. Table 7 compares the properties of TFC membranes formed via reverse IP technique with the conventionally made TFC membrane.

**Figure 12:**
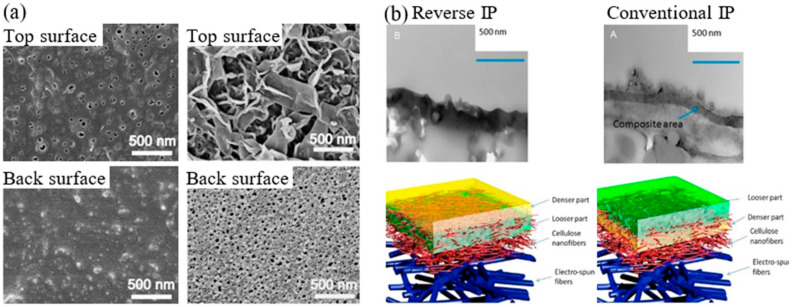
(**a**) SEM images of top and back surface of PA film formed via reverse IP and conventional IP [133] and (**b**) TEM (top) and schematic image (bottom) of reverse IP and conventional IP. The dense and loose parts of the PA layer are represented by yellow and green color, respectively [131].

To better understand the mechanism of PA synthesis, Yan et al. [133] compared the properties of the top and bottom surface of PA layer fabricated by using conventional IP over those constructed by using the reverse IP technique. They found that the amine monomers moved from the aqueous phase towards the organic/newly formed interface, regardless of the IP technique, and concluded that the IP reaction was partial towards the organic interface. Nevertheless, since the organic solution is in direct contacted with the substrate in the reverse IP, crosslinked film can be developed from the top aqueous solution towards the substrate at the bottom. As a result, pores on the top surface of PA film are formed, instead of the typical ridge-and-valley structure in a conventional PA film (Figure 12a). The back surface of the PA polymerized via the reverse IP was also highly distinct compared to that created by conventional IP technique, since the sandwiched organic phase in reverse IP prevents the PA film from growing freely. Separately, Wang et al. [131] reported that the reverse IP polymerized PA layer is denser on its top structure, as shown in Figure 12b. The top denser structure was due to primary bulk diffusion while the loose structure originated from secondary stepwise diffusion adjacent to the substrate surface. Compared to the conventional IP membrane (7.1 L/m^2^·h·bar permeance), the reverse IP membrane achieved higher permeance (9 L/m^2^·h·bar) at similar MgSO_4_ rejection (~98%) when a modified substrate was used.

Contradictory result, however, was reported by Shen et al. [132] in which they found that reverse IP formed PA layers with multiple defects due to the rapid evaporation of organic solvent. To solve this problem, they formed a gelatin interlayer on the hydrophilic electrospun PAN substrate prior to reverse IP process. An ultrathin (45 nm), crumpled and defect-free PA was subsequently, developed with the help of gelatin interlayer. The process depended on the synergistic interaction between gelatin and TMC that restricted the dispersion of TMC and further regulated the rising speed of n-hexane towards the aqueous phase. The gelatin-modified membrane made of reverse IP achieved 33.7 L/m^2^·h·bar PWP and 97.5% MgSO_4_ rejection, almost triple the flux of commercial NF270 membrane at similar salt rejection. The authors further demonstrated that when the hydrophilic PAN substrate without gelatin interlayer was used, the reverse IP approach yielded poor results (9 L/m^2^·h·bar PWP and 91% MgSO_4_ rejection).

To improve the efficiency of reverse IP, Song et al. [80] pretreated the PSf substrate with ethanol and hexane prior to polymerization process, in order to minimize the hydrolyzation of TMC from leftover water adsorbed on the substrate. Nevertheless, the pinhole defect persists as the main problem that lowers the degree of NaCl rejection of this membrane, compared to conventionally formed ones. Furthermore, the negatively affected water flux in the reverse IP formed membrane could be attributed to the crater-like PA structures that significantly lower the surface area.

### 3.8. Summary of New or Modified IP Techniques

The modified/new IP techniques documented in the literature have proven to be useful and advantageous for fabricating PA TFC/TFN membranes. Using these techniques, the composite membranes with desired characteristics such as improved PA morphology, higher water flux and enhanced antifouling properties were able to be developed. For example, the ultrasound-based IP that forms a porous and looser PA layer could significantly enhance the membrane water permeability, while the support-free IP could make the resultant PA layer highly smooth for potential reduction in fouling propensity. These improvements are vital in the industrial applications to achieve better process efficiency. Many of these techniques could also reduce the chemical wastage and address the poor nanomaterial/monomer dispersion issue that trouble the fabrication of current TFC/TFN membranes. In particular, the use of spray- and electrospray-based IP, as well as the DSC technique (in support-free IP), could drastically reduce the amount of chemicals used during membrane fabrication. However, proper attention should be paid to the long-term stability and nanomaterial leaching issues of these novel membranes prior to wide-scale application and commercialization. In the following section, we discuss further the potential issues faced by these IP techniques.

## 4. Technical Challenges of New or Modified IP Techniques

Table 8 details the advantages and disadvantages of each IP technique, based on the thorough review on the new or modified IP techniques to fabricate TFC/TFN membrane in the previous section. Although the support-free IP technique such as DSC and IFIP are highly scalable, the main challenge lies in the transfer of fragile PA layer onto the substrate. In addition, this technique produces membranes with low interfacial stability due to the absence of mechanical interlocking between the substrate and the PA layer. To overcome this, it was suggested to use vacuum filtration/suction to enhance the interfacial adhesion of the PA layer [82]. However, the integration of both techniques might result in a low scalability of the overall technique especially in the final vacuum filtration step.

Despite exhibiting promising results in depositing 2D nanomaterial, filtration-based IP is unsuitable for the deposition of 3D nanomaterials; especially those that are smaller than the substrate pores (typically with molecular weight cutoff (MWCO) of 30–50 kDa [58]). The vacuum filtration may result in an extremely low or total absence of nanomaterials on the PA layer, as they could be filtered out or trapped within substrate pores. As a consequence, the effectiveness of such TFN membranes may be reduced. Both filtration- and spin-based IP are not easy to be scaled up as the commercially available machines are unable to handle large size of typical membrane sheet required (1 m in length) to form spiral wound membrane module. Compared to other novel IP technique, spin-based IP has another major problem, i.e., inevitable wastage of chemical and nanomaterial during spinning process. This method may face difficulties in mass production.

For ultrasound-based IP, only limited studies were documented in the literature. As this technique is relatively new, there is limited understanding on its fundamental knowledge. Spray-based IP meanwhile, is more competitive and can be readily integrated with existing membrane manufacturing lines, as long as long-term membrane stability and economic analysis have been carried out. Similarly, while electrospray-based IP demonstrates high potential in membrane fabrication due to its minimum use of chemicals and the precise control of PA thickness, this approach is quite time consuming (>1 h is needed to develop PA layer [128]). The use of high voltage equipment also warrants a high energy cost and poses an additional risk factor to the safety of the operators.

**Figure 13:**
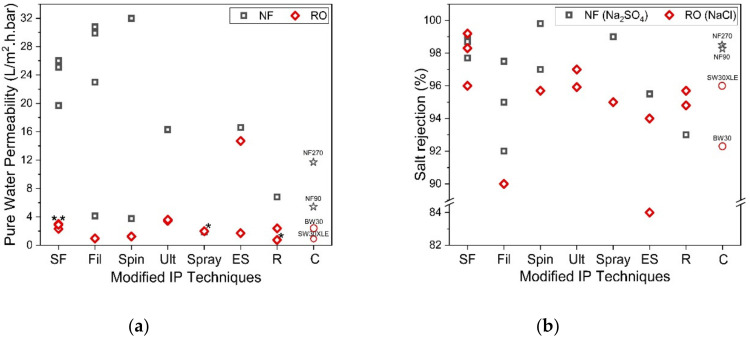
(**a**) Comparison of membrane permeability fabricated by using support-free IP (SF) [63,76,77,81,82,83], filtration-based IP (Fil) [29,87,88,89,90], spin-based IP (Spin) [30,97,98], ultrasound-based IP (Ult) [101,102], spray-based IP (Spray) [112,113], electrospray-based IP (ES) [31,126,127], reverse IP (R) [80,129,130] and commercial membrane (C) and (**b**) Comparison of salt rejection (Na_2_SO_4_ for NF and NaCl for RO) for each novel IP technique. (* Note: The data were obtained from water permeability of 2000 ppm NaCl solution).

**Figure 14:**
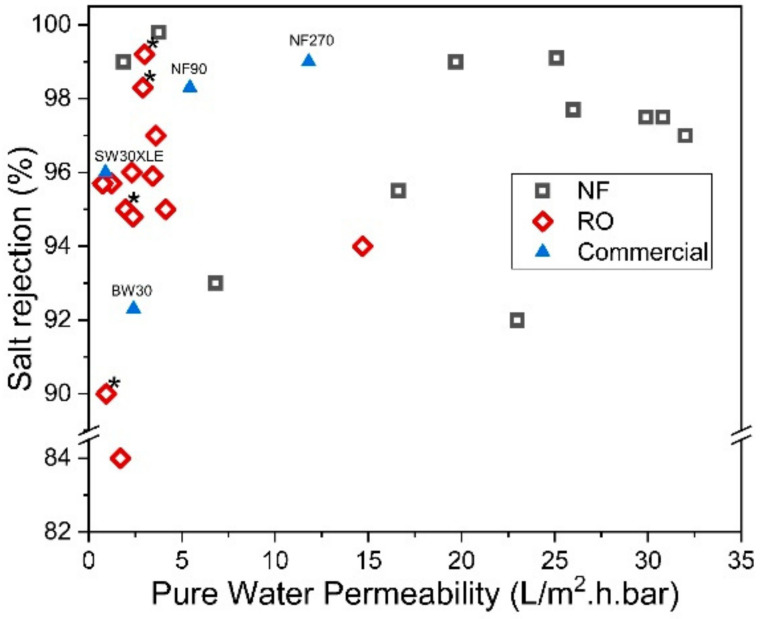
Salt rejection (Na_2_SO_4_ for NF and NaCl for RO) and water permeability of membranes made of novel IP techniques. (* Note: The data were obtained from water permeability of 2000 ppm NaCl solution).

On the other hand, the reverse IP procedure may prove to be rather similar to the conventional IP technique, except that the sequences of aqueous and organic solutions are reversed during the IP process. Reverse IP is particularly unsuitable for the fabrication of PA on hydrophilic substrates (e.g., cellulose nanofibers [131] and PAN nanofibers [132]); as such, pretreatment is required to modify substrate properties. This makes the overall membrane production complex and may incur additional manufacturing costs. Similar to conventional IP, the reverse IP approach also encounters difficulties in controlling the film properties (e.g., PA thickness, roughness, porosity and surface chemistry). This is due to their limiting fabrication procedures that self-terminate (diffusion-limited), leading to an uncontrolled PA growth. Figure 13 compares the PWP and degree of salt rejection of membranes made by novel/modified IP technique with conventionally made commercial NF/RO membranes, while Figure 14 shows the relationship between the membrane salt rejection and its water permeability. As can be seen, the novel/modified IP techniques, in general, can potentially overcome the trade-off effect in the conventional membranes, by further increasing the water permeability, without compromising salt rejection.

## 5. Conclusions and Future Outlook

Conventional IP technique is the dominant technology for the fabrication of PA layers in many commercial TFC-NF and -RO membranes, as well as for the development of TFC or TFN membranes for various lab-scale studies. This technique, however, has several challenges which somewhat limit its wider domain of applications. It is associated with the loss, as well as uneven distribution, of nanomaterials within/on the PA layer and the existence of a relatively thick selective layer which impedes a high degree of water permeation. Herein, this paper reviewed the progress of various novel/modified IP techniques for the fabrication of advanced TFC/TFN membranes in both pressure-driven and osmotically driven processes.

Compared to the typical fabrication method, the novel/modified IP approaches are superior in fabricating membranes with greater filtration performance owing to the enhanced PA chemistry (i.e., crosslinking degree) and morphology (i.e., pore size, thickness and smoothness). New TFC/TFN membranes made by support-free IP demonstrate higher fouling resistance due to the smoother formed PA layer, while the filtration-based IP-embedded nanofillers in TFN membranes avert leaching issues and ensure the membrane surface integrity. With precise control of shearing force, spin-based IP can enhance uniformity and performance of the PA layer. Ultrasound-based IP conversely forms loosely packed PA with larger free volume that counters the water flux–salt rejection trade-off effect. Other IP approaches, for instance, the spray-based and electrospray-based IPs produce PA films requiring minimal usage of chemicals.

Despite substantial improvements in existing novel IP approaches, future studies must focus on resolving their downsides, which hinder their lab- and commercial-scale implementation. Among the prominent issues that warrant further attention of the scientific community include the significant loss of materials (spin-based IP), difficulty in transferring the PA structure to the substrate (support-free IP), relatively long fabrication time (electrospray-based IP) and managing the uniform thickness of PA layer (filtration-based IP). There is still much room in improving current novel/modified IP processes even though the lab-scale membranes fabricated by using these novel/modified IP techniques have consistently shown more promising results over membranes made by conventional ones.

The afore-suggested concepts should account the economics and environmental impact of IP-based PA thin film preparation for water-filtration membranes. For one, expediting the fabrication process through fewer and/or simpler steps would be a large plus point in lowering the cost of membranes and producing cost-accessible water-filtration systems. Second, greener “facial” modified IP techniques through the use of renewable resources should be encouraged to make the membrane fabrication process eco-friendlier and sustainable. We hope this review article could provide insights into the development of effective IP techniques for TFC/TFN membrane fabrication for enhanced water separation.

## Figures and Tables

**Table 1:** Comparison of TFC membranes fabricated by using conventional and support-free IP techniques.

Year	Authors	Support-Free IP Conditions	Application	*^a^* Performance Comparison		Unique PA Structure
				Conventional IP	Support-Free IP (Optimum Membrane)	
**2016**	Karan et al. [32]	Immersion of nanostrand coated XP84 substrate into 3 wt % MPD followed by 0.15 wt % TMC. Nanostrand interlayer was removed via acid dissolution or HCl generated from IP reaction.	OSN	Commercial membrane (DuraMem DM150) Methanol permeance: ~0.48 L/m^2^·h·bar Acetonitrile permeance: 0.47 L/m^2^·h·bar	Methanol permeance: 52.2 L/m^2^·h·bar Acetonitrile permeance: 112 L/m^2^·h·bar Methyl orange rejection: 98.9%	-Crumpled/ridge-and-valley structures observed -Ultrathin PA layer (~8 nm)
**2017**	Park et al. [75]	Support-free PA was crosslinked between 3 wt % MPD and 0.1 wt % TMC. It was followed by drainage of excess solutions for attachment onto hydrolyzed polyacrylonitrile (PAN50) substrate.	RO	Permeability: 0.81 L/m^2^·h·bar (NaCl) NaCl rejection: 95.7% Zeta potential: −29.2 mV CA: 69.8°	Permeability: 0.86 L/m^2^·h·bar (NaCl) NaCl rejection: 99% Zeta potential: −22.3 mV CA: 67.2°	-Defect-free, thinner and smoother PA structure -Absence of typical ridge-and-valley structures (only nodules were formed)
**2017**	Park et al. [34]	Support-free PA was developed by using 0.025 wt % MPD and 0.1 wt % TMC. Both monomer solutions were spread through a slot die nozzle) followed by self-attachment onto hydrolyzed PAN50 substrate.	RO	Permeability: 1.55 L/m^2^·h·bar (NaCl) NaCl rejection: 68.7% Zeta potential: ~-29.5 mV CA: 71.8°	Permeability: 2.05 L/m^2^·h·bar (NaCl) NaCl rejection: 99.1% Zeta potential: ~-23.1 mV CA: 65.8°	-Smoother PA with ultrathin layer (~9 nm) -Absence of typical ridge-and-valley structure -Higher crosslinking degree of PA layer
**2017**	Cui et al. [76]	Formation of support-free PA, using 2 wt % MPD and 0.1 wt % TMC. Excess solution was drained after >5 h. The PA layer supported by track-etched membrane or non-woven fabric was then post-treated in DMF solution	FO	*n/a*	*J_v_*: ~6.2 L/m^2^.h *J_s_*: ~0.12 g/m^2^.h	-Typical ridge-and-valley structure formed
			RO	Commercial membrane (Dow SW30XLE) PWP *^b^*: 0.7 L/m^2^·h·bar NaCl rejection *^b^*: 99.7%	PWP: 2.31 L/m^2^·h·bar NaCl rejection: 96%	
**2018**	Zhu et al. [77]	Support-free PA was established with 0.025 wt % PIP and 0.05 wt % TMC followed by filtration of aqueous solution through PAN400C substrate	NF	Commercial membrane (Sepro NF 2A) [78] PWP: 10.1 L/m^2^·h·bar NaCl rejection: 24.8%	PWP: 25.1 L/m^2^·h·bar Na_2_SO_4_ rejection: 99.1% NaCl rejection: 28%	- Extremely thin PA layer (12 nm) - PA with sparse volcano-like structure was obtained
**2018**	Jiang et al. [79]	Formation of support-free PA (3 wt % MPD and 0.15 wt % TMC) followed by floating nanofilm on water surface and manual attachment onto PSf support membrane	RO	Commercial membrane (Dow SW30XLE) PWP *^b^*: 0.71 L/m^2^·h·bar NaCl rejection *^b^*: 99.7%	Permeability: 2.69 L/m^2^·h·bar (NaCl) NaCl rejection: 96%	-Ultrathin PA layer (~6 nm) -Formation of nodules that are similar to typical NF membranes.
**2018**	Trivedi et al. [74]	Formation of support-free PA (0.05 wt % PEI and 0.05 wt % TMC) followed by manual attachment onto polyethersulfone (PES) support membrane	NF	Permeability: ~20 L/m^2^·h·bar (Na_2_SO_4_) Na_2_SO_4_ rejection: ~85% NaCl rejection: ~27%	Permeability: ~20 L/m^2^·h·bar (Na_2_SO_4_) Na_2_SO_4_ rejection: ~82% NaCl rejection: ~30%	-Thin PA layer formed (~25 nm) -PA layer with similar roughness and thickness was obtained
**2019**	Song et al. [80]	Formation of support-free PA (2 wt % MPD and 0.1 wt % TMC) followed by filtration of aqueous solution through PSf substrate	RO	Permeability: ~1.55 L/m^2^·h·bar (NaCl) NaCl rejection: ~99%	Permeability: 0.94 L/m^2^·h·bar (NaCl) NaCl rejection: 96.4%	- PA layer with significantly smoother surface was achieved -Absence of typical ridge-and-valley structures.
**2019**	Zhang et al. [81]	Formation of support-free PA (0.6 wt % PIP and 0.025 wt % TMC) followed by drainage of excess solutions for attachment onto PES substrate	NF	PWP: ~16.3 L/m^2^·h·bar Na_2_SO_4_ rejection: ~99% MgCl_2_ rejection: ~94%	PWP: ~19.7 L/m^2^·h·bar Na_2_SO_4_ rejection: ~99% MgCl_2_ rejection: ~94%	-Distinct boundary between PA and support membrane -No SMPB observed
				(After ethanol immersion) PWP: ~18.7 L/m^2^·h·bar Na_2_SO_4_ rejection: ~98% MgCl_2_ rejection: ~90%	(After ethanol immersion) PWP: ~260 L/m^2^·h·bar Na_2_SO_4_ rejection: <5% MgCl_2_ rejection: <5%	
**2020**	Park and Lee [82]	Formation of support-free PA (0.025 wt % MPD and 0.1 wt % TMC, both spread through a slot die nozzle) followed self-attachment onto modified PSf support membrane	RO	Commercial membrane (Nitto SWC4+) Permeability: 1.6 L/m^2^·h·bar NaCl rejection: 99.2%	Permeability: 3 L/m^2^·h·bar (NaCl) NaCl rejection: 99.2%	-Ultrathin PA layer (~7 nm)
**2020**	Jiang et al. [63]	Formation of support-free PA via microscale dispersion of 0.05 wt % TMC onto modified PSf support membrane with unremoved residual PIP (0.025 wt %)	NF	Commercial membrane (Dow NF270) PWP *^b^*: ~12.07 L/m^2^·h·bar MgSO_4_ rejection *^b^*: >97%	Permeability: ~26.6 L/m^2^·h·bar (Na_2_SO_4_) Na_2_SO_4_ rejection: 98.7%	-Ultrathin PA layers -Smooth PA with slight nodular structures
		Formation of support-free PA via microscale dispersion of 0.05 wt % TMC onto modified PSf support membrane with unremoved residual MPD (0.025 wt %)	RO	Commercial membrane (Dow SW30XLE) PWP *^b^*: 0.7 L/m^2^·h·bar NaCl rejection *^b^*: 99.7%	Permeability: 2.9 L/m^2^·h·bar (NaCl) NaCl rejection: 98.3%	
**2020**	Ma et al. [83]	Formation of PA on hexane–jelly interface (0.02 wt % PIP in jelly and 0.07 wt % TMC in hexane). The jelly was then dissolved and support-free PA was manually attached to the PES substrate via vacuum filtration adhesion	NF	Commercial membrane (Dow NF270) PWP *^b^*: ~12.07 L/m^2^·h·bar MgSO_4_ rejection *^b^*: >97%	Permeability: ~26 L/m^2^·h·bar (Na_2_SO_4_) Na_2_SO_4_ rejection: 97.7%	-PA layer with thinner and smoother structure was developed

*^a^* Jv, water flux; Js, reverse salt flux; PWP, pure water permeability; CA, contact angle. *^b^* The commercial NF/RO membrane data were obtained from Dow’s Product Data Sheet and inserted in the table for the work that did not study control membrane (made of conventional IP technique).

**Table 2:** Comparison of TFN membranes fabricated by using conventional and filtration-based IP.

Year	Authors	Filtration IP Conditions	Application	*^a^* Performance Comparison		*^a^* Unique PA Structure
				Conventional IP	Filtration IP (Optimum Membrane)	
**2017**	Wu et al. [90]	Filtration of 0.1 wt % PIP containing 5 mg attapulgite through PES substrate followed by contact with 1 wt % TMC	NF	Commercial NF membrane (Sepro NF 2A) [78] PWP: 10.1 L/m^2^·h·bar NaCl rejection: 24.8%	PWP: 23 L/m^2^·h·bar Na_2_SO_4_ rejection: 92% FRR: 95.7% (tested with 1 g/L humic acid for 42.5 h)	- Even nanomaterial distribution - Rough PA layer (1.37 roughness area ratio)
**2018**	Al Aani et al. [29]	Filtration of 2 wt % MPD through metal oxide/CNT-coated (0.0025 mg/cm^2^) PES substrate followed by contact with 0.1 wt % TMC	RO	Commercial RO membrane (Dow SW30XLE) PWP *^b^*: 0.7 L/m^2^·h·bar NaCl rejection *^b^*: 99.7%	PWP: >0.95 L/m^2^·h·bar NaCl rejection: >90%	- Even nanomaterial distribution - Smooth PA layer (R_a_: ~10 nm) - Increased hydrophilicity
**2019**	Lai et al. [87]	Filtration of 2 wt % PIP through GO-coated (0.03 g/m^2^) PSf substrate followed by contact with 0.2 wt % TMC	NF	PWP: 1.80 L/m^2^·h·bar CA: ~46° Na_2_SO_4_ rejection: >95% Flux decline: 24% (tested 0.5 g/L BSA for 4 h)	PWP: 4.13 L/m^2^·h·bar CA: ~30° Na_2_SO_4_ rejection: >95% Flux decline: 1.1% (tested 0.5 g/L BSA for 4 h)	- Smoother PA layer formed - Thin PA layer (53 nm) - Low crosslinking degree (63.5%)
**2019**	Zhu et al. [88]	Filtration of 0.2 wt % PIP containing 20.5 µg/cm^2^ UiO-66-NH_2_ through PAN substrate followed by contact with 0.15 wt % TMC	NF	Commercial NF membrane (Dow NF270) PWP *^b^*: ~12.07 L/m^2^·h·bar MgSO_4_ rejection *^b^*: >97%	PWP: 30.8 L/m^2^·h·bar Na_2_SO_4_ rejection: 97.5% NaCl rejection: 20%	- Even nanomaterial distribution - Rough PA layer (R_a_: 55 nm) - Increased hydrophilicity
**2019**	Ren et al. [89]	Filtration of 0.1 wt % PIP containing 0.02 wt % *o*-POPs through PAN substrate followed by contact with 0.1 wt % TMC	NF	Commercial NF membrane (Dow NF270) PWP *^b^*: ~12.07 L/m^2^·h·bar MgSO_4_ rejection *^b^*: >97%	PWP: 29.9 L/m^2^·h·bar Na_2_SO_4_ rejection: 97.5%	- Even nanomaterial distribution - Crumpled and rough PA layer

*^a^* PWP, pure water permeability; CA, contact angle; FRR, flux recovery ratio; R_a_, average roughness. *^b^* The commercial NF/RO membrane data were obtained from Dow’s Product Data Sheet and inserted in the table for the work that did not study control membrane (made of conventional IP technique).

**Table 3:** Comparison of TFC/TFN membranes fabricated by using conventional and spin-based IP.

Year	Authors	Spin IP Conditions	Application	*^a^* Performance Comparison		Unique PA Structure
				Conventional IP	Spin-Based IP (Optimum Membrane)	
**2012**	An et al. [95]	Immersion of modified PAN substrate in 0.1 wt % 1,3-diaminopropane followed by spin removal of 0.2 wt % succinyl chloride at 6000 rpm	Pervaporation	Permeate flux *^b^*: ~375 g/m^2^·h Ethanol rejection: 93.6% Ethanol permeability: ~12 × 10^−4^ g/m.h.MPa CA: ~80°	Permeate flux *^b^*: ~660 g/m^2^·h Ethanol rejection: 99.3% Ethanol permeability: ~1.4 × 10^−4^ g/m.h.MPa CA: ~58°	- Parallel lines formed contributed to increased roughness - 46% thinner PA layer - Denser PA layer with smaller cavities
**2018**	Yuan et al. [97]	Immersion of PES substrate in 0.5 wt % PIP followed by spinning at 3000 rpm for 40 s. Substrate was then contacted with 0.03 wt % NTSC before drying through spinning, marking the end of 1 cycle (5 cycles is optimal)	RO	Permeability: 2.21 L/m^2^·h·bar (NaCl) MgSO_4_ rejection: 82.04% CaCl rejection: 73.5% NaCl rejection: 58.2% CA: ~68°	Permeability: 1.24 L/m^2^·h·bar (NaCl) MgSO_4_ rejection: 98.7% CaCl rejection: 98.2% NaCl rejection: 95.7% CA: ~68°	- Linear increase of PSA thickness per layer (2.72 nm/layer) - Minimal change in roughness
**2018**	He et al. [98]	Immersion of PES substrate in 0.5 wt % PIP followed by spinning at 3500 rpm for 30 s. Substrate was then contacted with 0.05 wt % TCSP before drying through spinning, marking the end of 1 cycle (5 cycles is optimal)	NF	PWP: 1.49 L/m^2^·h·bar Na_2_SO_4_ rejection: 98.3% MgSO_4_ rejection: 92.92%	PWP: 3.75 L/m^2^·h·bar Na_2_SO_4_ rejection: 99.8% MgSO_4_ rejection: 99.37%	- Thinner PSA layer (80 vs. 138 nm) - Minimal change in roughness
**2020**	Kang et al. [30]	Spin removal of 0.5 wt % PIP on GO-coated (6 mg/m^2^) nylon substrate at 600 rpm for 40 s followed by contact with 0.5 wt % TMC	NF	Commercial NF membrane (Dow NF270) PWP *^c^*: ~12.07 L/m^2^·h·bar MgSO_4_ rejection *^c^*: >97%	PWP: ~32 L/m^2^·h·bar Na_2_SO_4_ rejection: ~97% MgSO_4_ rejection: ~80%	- Extremely thin PA layer (20–35 nm) - Uniform monomer distribution

*^a^* PWP, pure water permeability; CA, contact angle. *^b^* Tested with 90 wt % aqueous ethanol solution. *^c^* The commercial NF membrane data were obtained from Dow’s Product Data Sheet and inserted in the table for the work that did not study control membrane (made of conventional IP technique).

**Table 4:** Comparison of TFC membranes fabricated by using conventional and ultrasound-assisted IP.

Year	Authors	Ultrasound IP Conditions	Application	*^a^* Performance Comparison		*^a^* Unique PA Structure
				Conventional IP	Ultrasound-Assisted IP (Optimum Membrane)	
**2019**	Shen at al. [101]	Immersion of PSf substrate in 2.0 wt % MPD followed by contact with 0.1 wt % TMC under an ultrasonication circumstance (40 kHz and 360 W)	FO	*J_v_*: ~12 L/m^2^·h *J_s_*: ~4.6 g/m^2^·h	*J_v_*: ~32.5 L/m^2^·h *J_s_*: ~4.3 g/m^2^·h	- Rougher PA layer formed - Thicker PA layer albeit less dense due to the larger cavities formed - Higher crosslinking degree achieved
			PRO	*J_v_*: ~25 L/m^2^·h *J_s_*: ~9 g/m^2^·h	*J_v_*: ~52 L/m^2^·h *J_s_*: ~7.3 g/m^2^·h	
			RO	PWP: 1.99 L/m^2^·h·bar NaCl rejection: 94.72% Selectivity (*B/A* ratio): 0.09 bar	PWP: 3.44 L/m^2^·h·bar NaCl rejection: 95.92% Selectivity (*B/A* ratio): 0.07 bar	
**2019**	Shen at al. [101]	Immersion of PSf substrate in 0.35 wt % PIP followed by contact with 0.15 wt % TMC under an ultrasonication circumstance (40 kHz and 360 W)	NF	PWP: 7.5 L/m^2^·h·bar NaCl rejection: 27.5%	PWP: 16.3 L/m^2^·h·bar NaCl rejection:30.0%	*n/a*
**2020**	Shen et al. [102]	Immersion of PSf substrate in 2.0 wt % MPD followed by contact with 0.1 wt % TMC for 1 min under an ultrasonication circumstance (60 kHz and 480 W)	FO	*J_v_*: ~25 L/m^2^·h *J_s_*: ~10.4 g/m^2^·h CA: 80° FRR *^b^*: 83.3%	*J_v_*: ~75 L/m^2^·h *J_s_*: ~8 g/m^2^·h CA: 55° FRR *^b^*: 97.0%	- Rougher (R_a_: 90 nm) and thicker PA layer formed - Higher crosslinking degree achieved - PA layer showed increased resistance against gypsum scaling
			PRO	*J_v_*: ~43 L/m^2^·h *J_s_*: ~19.5 g/m^2^·h	*J_v_*: ~120 L/m^2^·h *J_s_*: ~12 g/m^2^·h	
			RO	PWP: 1.9 L/m^2^·h·bar NaCl rejection: ~94.2% Selectivity (*B/A* ratio): ~0.1 bar	PWP: 3.6 L/m^2^·h·bar NaCl rejection: ~97% Selectivity (*B/A* ratio): ~0.04 bar	

*^a^ J_v_*, water flux; *J_s_*, reverse salt flux; PWP, pure water permeability; *B*, salt permeability; *A*, water permeance; CA, contact angle; FRR, flux recovery ratio; R_a_, average roughness. *^b^* Measured after testing with gypsum for 18 h, followed by membrane cleaning.

**Table 5:** Comparison of TFC membranes fabricated by using conventional and spray-based IP.

Year	Authors	Spray IP Conditions	Application	Performance Comparison		Unique PA Structure
				Conventional IP	Spray-Based IP (Optimum Membrane)	
**2013**	Tsuru et al. [112]	Immersion of PSf substrate in 2 wt % MPD followed by spraying of 0.05 wt % TMC, using airbrush (30 mg/s flow rate for 20 s). Then, 0.1 wt % TMC was allowed to contact with the membrane.	RO	Permeance: ~1.14 L/m^2^·h·bar (NaCl) NaCl rejection: >95% Glucose rejection: >95% Ethanol rejection: ~40%	Permeance: ~1.98 L/m^2^·h·bar (NaCl) NaCl rejection: >95% Glucose rejection: >95% Ethanol rejection: ~45%	- Multilayered large and small ridge-and-valley structure formed - Higher crosslinking degree as spray time increases
**2017**	Shan et al. [33]	Spraying 1.25 wt % PEI followed by spraying 0.15 wt % TMC on PSf substrate at 2 mL/s flow rate. Each layer was sprayed by 5 s to achieve 5 layers.	NF	Permeance *^a^*: 5.3 L/m^2^·h·bar	Permeance *^a^*: 124.6 L/m^2^·h·bar Humic acid rejection: 99.3%	- Extremely thin PA layer formed (25 nm)
**2019**	Morales-Cuevas et al. [113]	Brushing aqueous solution (0.25 wt % PIP, 0.25 wt % PVA and 0.5 wt % NaOH) onto PSf substrate followed by the spraying 1 wt % TMC solution (5 mL)	NF	PWP: 1.23 L/m^2^·h·bar Na_2_SO_4_ rejection: ~99% NaCl rejection: ~20%	PWP: 1.87 L/m^2^·h·bar Na_2_SO_4_ rejection: 99% NaCl rejection: ~40%	- Smoother PA layer (Average roughness: 48 nm)

*^a^* The data were collected from membranes evaluated by using natural water from the Miyun reservoir (Beijing).

**Table 6:** Comparison of TFC membranes fabricated by using conventional and electrospray IP.

Year	Authors	Electrospray IP Conditions	Application	*^a^* Performance Comparison		*^a^* Unique PA Structure
				Conventional IP	Electrospray IP (Optimum Membrane)	
**2018**	Chowdhury et al. [31]	Electrospraying 0.083 wt % MPD and 0.05 wt % TMC onto a PAN substrate-mounted rotating drum (flow rate: 5 mL/h, tip to drum distance: 2.5–5 cm and rotating speed: 20 rpm)	RO	Commercial RO membrane (SW30XLE) PWP *^b^*: 0.7 L/m^2^·h·bar NaCl rejection *^b^*: 99.7% RMS: ~84 nm	PWP: 14.7 L/m^2^·h·bar NaCl rejection: 94% RMS: 13.4 nm	- Extremely thin PA layer (25 nm) with high repeatability - Extremely smooth PA layer
**2018**	Ma et al. [126]	Electrospraying 2.0 wt % MPD and 0.2 wt % TMC onto a PES substrate-mounted rotating drum (flow rate: 1.2 mL/h, tip to drum distance: 6 cm and rotating speed: 100 rpm)	RO	PWP: 0.55 L/m^2^·h·bar CA: 53.3°	PWP: 1.7 L/m^2^·h·bar NaCl rejection: 84% Na_2_SO_4_ rejection: 94% CA: 72.0°	- Linear PA growth rate (~1 nm/min) - Extremely smooth (R_a_: 1.2 nm) and thin PA layer (~30 nm)
**2020**	Yang et al. [127]	Electrospraying 0.24 wt % PIP and 0.08 wt % TMC onto a PES substrate-mounted rotating drum (flow rate: 1.2 mL/h, tip to drum distance: 6 cm and rotating speed: 80 rpm)	NF	PWP: 4.4 L/m^2^·h·bar Na_2_SO_4_ rejection: 98.1%	PWP: 16.6 L/m^2^·h·bar Na_2_SO_4_ rejection: 95.5%	- Linear PA growth rate (~0.33 nm/min) - Extremely smooth (R_a_: 15.3 nm) and thin PA layer (22 nm) - Lamellar PA layer that can provide extra water channels

*^a^* PWP, pure water permeability; CA, contact angle; RMS, root mean square roughness; R_a_, average roughness. *^b^* The commercial NF membrane data were obtained from Dow’s Product Data Sheet and inserted in the table for the work that did not study control membrane (made of conventional IP technique).

**Table 7:** Comparison of TFC membranes fabricated by using conventional and reverse IP.

Year	Authors	Reverse IP Conditions	Application	*^a^* Performance Comparison		Unique PA Structure
				Conventional IP	Reverse IP (Optimum Membrane)	
**2014**	Wang et al. [131]	Immersion of substrate (modified PAN on polyethylene terephthalate (PET)) into 0.1 wt % TMC followed by contacting with 3 wt % PIP.	NF	Permeability: 7.1 L/m^2^·h·bar (MgSO_4_) MgSO_4_ rejection: ~99% MgCl_2_ rejection: ~99%	Permeability: 9.0 L/m^2^·h·bar (MgSO_4_) MgSO_4_ rejection: ~98% MgCl_2_ rejection: ~97%	- Dense part of PA layer was formed on the top instead of near the substrate as in conventional IP
**2016**	Mahdavi and Moslehi [129]	Immersion of substrate (PET) into 0.3 wt % TMC followed by contacting with 1 wt % PPD.	NF	Commercial NF membrane (Sepro NF 2A) [78] PWP: 10.1 L/m^2^·h·bar NaCl rejection: 24.8%	PWP: 6.8 L/m^2^·h·bar NaCl rejection: 78% Na_2_SO_4_ rejection: 93%	- Smooth PA layer without defects formed on both electrospun and casted substrate
**2018**	Qanati et al. [130]	Immersion of substrate (polyvinylidene fluoride) into 0.05 wt % 1,2,4,5-benzene tetracarbonyl chloride and 0.05 wt % TMC followed by contacting with 2 wt % ethylenediamine and 2 wt % triethylamine.	RO	Commercial RO membrane (Dow SW30XLE) PWP *^b^*: 0.7 L/m^2^·h·bar NaCl rejection *^b^*: 99.7%	PWP: 2.38 L/m^2^·h·bar NaCl rejection: 94.8% NaCl rejection after chlorine test: 93.4%	- Polyimide selective layer shows similar structure as typical NF PA layer
**2019**	Shen et al. [132]	Immersion of substrate (gelatin on PAN) into 0.2 wt % TMC followed by contacting with 1 wt % PIP	NF	Commercial NF membrane (Dow NF270) PWP *^b^*: ~12.07 L/m^2^·h·bar MgSO_4_ rejection *^b^*: >97%	PWP: 33.7 L/m^2^·h·bar MgSO_4_ rejection: 97.5% NaCl rejection: 14.3%	-Ultrathin PA layer formed -Crumpled, defect-free PA observed
**2019**	Song et al. [80]	Immersion of substrate (PSf) into 0.1 wt % TMC followed by contacting with 2 wt % MPD	RO	Permeability: ~1.55 L/m^2^·h·bar (NaCl) NaCl rejection: ~99%	Permeability: ~0.75 L/m^2^·h·bar (NaCl) NaCl rejection: ~95.7%	-Crater-like/porous structures formed instead of typical ridge-and-valley structures -Smooth PA (Average roughness: 23 nm)

*^a^* PWP, pure water permeability. *^b^* The commercial NF membrane data were obtained from Dow’s Product Data Sheet and inserted in the table for the work that did not study control membrane (made of conventional IP technique).

**Table 8:** Advantages and disadvantages of novel IP techniques.

Technique	Advantages	Disadvantages
**Support-free IP**	- High scalability (DSC and IFIP) - High precision (automated DSC and IFIP) - Able to form PA at very low monomer concentration	- Difficult to transfer/attach PA film onto substrate
**Filtration-based IP**	- Suitable to deposit 2D nanosheets on the substrate - No leaching of nanomaterials during filtration - Nanomaterials can be well embedded within PA layer with good stability	- Not suitable for depositing 3D nanomaterials with particle size much smaller than substrate pore size - Precise control of PA layer thickness is rather difficult - Low scalability
**Spin-based IP**	- Rapid process - Able to produce highly uniform PA layer	- Low scalability - Chemical/nanomaterials wastage is unavoidable during spinning - Require precise control of shearing force
**Ultrasound-based IP**	-Formation of nanovoids within PA layer that could improve water flux	- Limited studies
**Spray-based IP**	- High scalability - Minimum use of chemicals/nanomaterials - Relatively fast process - Precise control of PA layer thickness	- Lack of long-term membrane stability evaluation - Lack of economic analysis
**Electrospray-based IP**	- Moderate scalability - Minimum use of chemicals - Precise control of PA layer thickness (at nm scale)	- Slow process (>1 h) - Relatively high energy requirement (high voltage equipment) - Difficult to produce large-sheet of membrane
**Reverse IP**	- Suitable for hydrophobic substrate	- Difficult to form defect-free TFC membrane, using widely used substrate (e.g., PSf and PAN)
